# New Respiratory Enterovirus and Recombinant Rhinoviruses among Circulating Picornaviruses 

**DOI:** 10.3201/eid1505.081286

**Published:** 2009-05

**Authors:** Caroline Tapparel, Thomas Junier, Daniel Gerlach, Sandra Van Belle, Lara Turin, Samuel Cordey, Kathrin Mühlemann, Nicolas Regamey, John-David Aubert, Paola M. Soccal, Philippe Eigenmann, Evgeny Zdobnov, Laurent Kaiser

**Affiliations:** University of Geneva Hospitals, Geneva, Switzerland (C. Tapparel, S. Van Belle, L. Turin, S. Cordey, P. M. Soccal, P. Eigenmann, L. Kaiser); University of Geneva Medical School, Geneva (C. Tapparel, T. Junier, D. Gerlach, S. Van Belle, L. Turin, S. Cordey, E. Zdobnov, L. Kaiser); Swiss Institute of Bioinformatics, Geneva (T. Junier, D. Gerlach, E. Zdobnov); University Hospital of Bern, Bern, Switzerland (K. Mühlemann, N. Regamey); University Hospital of Lausanne, Lausanne, Switzerland (J.-D. Aubert); Imperial College London, London, UK (E. Zdobnov); 1 These authors contributed equally to this article.

**Keywords:** Respiratory infections, molecular epidemiology, picornavirus, rhinovirus, enterovirus, recombination, capsid protein, nonstructural protein, genotype, research

## Abstract

Rhinoviruses and enteroviruses are leading causes of respiratory infections. To evaluate genotypic diversity and identify forces shaping picornavirus evolution, we screened persons with respiratory illnesses by using rhinovirus-specific or generic real-time PCR assays. We then sequenced the 5′ untranslated region, capsid protein VP1, and protease precursor 3CD regions of virus-positive samples. Subsequent phylogenetic analysis identified the large genotypic diversity of rhinoviruses circulating in humans. We identified and completed the genome sequence of a new enterovirus genotype associated with respiratory symptoms and acute otitis media, confirming the close relationship between rhinoviruses and enteroviruses and the need to detect both viruses in respiratory specimens. Finally, we identified recombinants among circulating rhinoviruses and mapped their recombination sites, thereby demonstrating that rhinoviruses can recombine in their natural host. This study clarifies the diversity and explains the reasons for evolution of these viruses.

## Abstract 2

Increased genomic diversity of these viruses is demonstrated.

Human rhinoviruses (HRVs) and enteroviruses (HEVs) are leading causes of infection in humans. These 2 picornaviruses share an identical genomic organization, have similar functional RNA secondary structures, and are classified within the same genus (www.ictvonline.org/virusTaxonomy.asp) because of their high sequence homology (*1*). However, despite their common genomic features, these 2 groups of viruses have different phenotypic characteristics. In vivo, rhinoviruses are restricted to the respiratory tract, whereas enteroviruses infect primarily the gastrointestinal tract and can spread to other sites such as the central nervous system. However, some enteroviruses exhibit specific respiratory tropism and thus have properties similar to rhinoviruses (*2*–*5*). In vitro, most HRVs and HEVs differ by their optimal growth temperature, acid tolerance, receptor usage, and cell tropism. The genomic basis for these phenotypic differences between similar viruses is not yet fully understood.

HRVs and HEVs are characterized by ≈100 serotypes. Recently, molecular diagnostic tools have shown that this diversity expands beyond those predefined serotypes and encompasses also previously unrecognized rhinovirus and enterovirus genotypes. As an example, a new HRV lineage named HRV-C was recently identified and now complements the 2 previously known A and B lineages (*6*–*8*) (N.J. Knowles, pers. comm.). The C lineage has not only a distinct phylogeny (*9*–*16*) but is also characterized by specific cis-acting RNA structures (*17*).

In this study, we screened a large number of persons with acute respiratory diseases by using assays designed to overcome the diversity of both rhinoviruses and enteroviruses circulating in humans. Whenever possible, we systematically sequenced 5′ untranslated region (UTR), capsid protein VP1, and protease precursor 3CD regions of strains. Our goals were 1) to characterize the diversity of circulating rhinoviruses and, to a lesser extent, enteroviruses, to identify putative new picornavirus variants, and 2) to assess whether recombination may drive HRV evolution, which has not been shown in natural human infections (*18*).

## Materials and Methods

### RNA Extraction, Reverse Transcription-PCR, and Real-Time PCR

Reverse transcription–PCR (Superscript II; Invitrogen, Carlsbad, CA, USA) was performed on RNA extracted by using the HCV Amplicor Specimen Preparation kit (Roche, Indianapolis, IN, USA), TRIzol (Invitrogen), or the QIAamp Viral RNA Mini kit (QIAGEN, Valencia, CA, USA). Real-time PCR specific for HRV-A, HRV-B, and HEV (*19*), and a generic panenterhino real-time PCR (forward primer 5′-AGCCTGCGTGGCKGCC-3′, reverse primer 5′-GAAACACGGACACCCAAAGTAGT-3′, and probe 5-FAM-CTCCGGCCCCTGAATGYGGCTAA-TAMRA-3′), were performed in several cohort studies (Table).

**Table:** Characteristics of screened study populations and respiratory samples, Switzerland*

Type of study (no. enrolled)	Age group	Patient characteristics	Years of study	Type of specimens	PCR	No. (%) positive	Reference
Respiratory infection in newborns (243)	<1 y	Nonhospitalized children with initial respiratory episode with cough	1999–2005	NPS	HRV-A and HRV-B specific real time for the first 203 and panenterhino for 40	36 (15)	(*20*)
Lower respiratory tract infection in hospitalized patients (147)	Adults	Mainly immunocompromised patients with lower respiratory tract complications and comorbidities	2001–2003	BAL, NPS	HRV-A and HRV-B specific real time	16 (11)	(*21*)
Acute respiratory tract infection in children (653)	<17 y	Nonhospitalized children with AOM or pneumonia	2004–2007	NPS	Panenterhino	121 (18)	(*22*) and ongoing study
Lower respiratory tract infection in hospitalized patients (485)	Adults	Mainly immunocompromised patients with lower respiratory tract complications and concurrent illnesses	2003–2006	BAL, NPS	Panenterhino	52 (11)	(*21*) and ongoing study
Acute respiratory tract infection in children (64)	<12 y	Children at an emergency department with fever and acute respiratory symptoms treated with antimicrobial drugs	2006–2007	NPS	Panenterhino	23 (36)	NP
Isolation in routine procedures (NA)	Children and adults	Hospitalized patients	1999–2008	BAL, NPS	HE culture isolation	NA	NP

*NPS, nasopharyngeal samples; HRV, human rhinovirus; BAL, bronchoalveolar lavage; AOM, acute otitis media; NP, not published; NA, not available; HE, human embryonic primary fibroblast cell line.

### Clinical Specimens

Picornavirus-positive samples were detected from patients enrolled in cohort studies in different regions of Switzerland during 1999–2008. The main characteristics of these populations, type of respiratory specimens, and screening methods are shown in the Table. The rhinovirus serotypes used for 3CD sequencing were obtained from the American Type Culture Collection (Manassas, VA, USA).

### PCR and Sequencing

Sequencing was performed directly from the clinical specimen except for samples selected by routine isolation methods on human embryonic (HE) primary fibroblast cell lines (Table) or for HRV reference serotypes. Primers used to amplify the 5′-UTR and the VP1 and 3CD regions are listed in Technical Appendix 1 Table 1A.

Full-length genome sequences of CL-1231094, a related clinical strain of enterovirus, and partial sequences of CL-Fnp5 and CL-QJ274218 were obtained as follows. RNA extracted by using the QIAamp Viral RNA Mini kit (QIAGEN) plus DNase treatment or with Trizol was reverse transcribed with random-tagged primer FR26RV-N and amplified with the SMART RACE cDNA Amplification kit (Clontech, Mountain View, CA, USA) with a specific forward primer and FR20RV reverse primer (Technical Appendix 1 Table 1B) (*23*). Amplification products were separated by electrophoresis on agarose gels and fragments (0.6–2.5 kb) were extracted by using the QIAquick Gel Extraction kit (QIAGEN). Purified products were cloned by using the TOPO TA cloning kit (Invitrogen).

Minipreps were prepared from individual colonies and clones with the largest inserts were chosen for sequencing. Sequences obtained were used to design a new forward primer (Technical Appendix 1 Table 1) to advance toward the 3′ end of the genome. PCR products of 3′ genomic ends were obtained by using the BD Smart Race cDNA amplification kit (Becton Dickinson, Franklin Lakes, NJ, USA) according to manufacturer’s instructions. All PCR products were purified by using microcon columns (Millipore, Billerica, MA, USA) and sequenced by using the ABI Prism 3130XL DNA Sequencer (Applied Biosystems, Foster City, CA, USA). Chromatograms were imported for proofreading with the vector NTI Advance 10 program (Invitrogen). Overlapping fragments were assembled with the contigExpress module of the vector NTI Advance 10.

### Sequence Analysis, Phylogeny, and Bootscanning of Recombinants

Alignments were constructed by using MUSCLE (*24*) with a maximum of 64 iterations. (For detailed analyses, see http://cegg.unige.ch/picornavirus.) Multiple FastA was converted into PHYLIP format (for tree building) with the EMBOSS program Seqret (*25*). Trees were built with PhyML (*26*) by using the general time reversible model, BIONJ for the initial tree, and optimized tree topology and branch lengths. Trees with <50 species and larger trees used 16 and 8 rate categories, respectively. Transition/transversion ratios, proportions of invariant sites, and shape parameters of the γ distribution were estimated.

**Figure 1:**
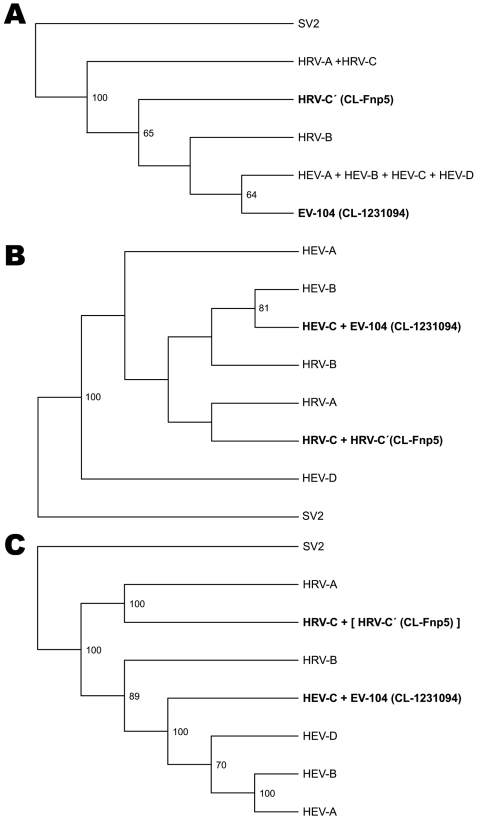
5′ untranslated region (A), capsid protein VP1 (B), and complete genome (C) phylogeny of the virus clades studied. Trees were produced by condensing the full phylogeny shown in Technical Appendix 2 Figure 1, panels A, B, and D. Human rhinovirus C′ (HRV-C′) includes the divergent rhinoviruses described in 2007 (*13*) and a related clinical strain (CL-Fnp5). HRV-C includes the new clade described since 2006 (*9*–*14*,*16*). Enterovirus 104 (EV-104) and the related strain CL-1231094 refer to a previously unknown enterovirus clade described in this study. In panel C, HRV-C′ is shown in brackets to indicate its expected location (based on VP1 and 3D sequences). Simian picornavirus 1 (SV2) was used as an outgroup. HEV, human enterovirus. Bootstrap support values <50 are not shown in the trees. New viruses are shown in **boldface**.

To investigate the hypothesis of recombination and map the breakpoints, we adapted the bootscanning method (*27*) as follows. The alignment was sliced into windows of constant size and fixed overlap and a 100-replicate maximum-likelihood (using HRV-93 as an outgroup) was computed for each window. From each tree, the distance between the candidate recombinant and all other sequences was extracted. This extraction yielded a matrix of distances for each window and for each alignment position. A threshold was defined as the lowest distance plus a fraction (15%) of the difference between the highest and lowest distances. The nearest neighbors of the candidate recombinant were defined as sequences at a distance smaller than this threshold. This distance ensured that the nearest neighbor, as well as any close relative, was always included. Possible recombination breakpoints thus corresponded to changes of nearest neighbors. Serotypes included in this analysis represented serotypes close to CL-013775 and CL-073908 on the basis of 5′-UTR and VP1 phlyogenetic trees (Technical Appendix 2 Figure 1, panels A, B), as well as serotypes close to CL-135587 on the basis of VP1 and 3CD phlyogenetic trees (Technical Appendix 2 Figure 1, panels B, C) and whose full-length sequence was available.

Distance matrices were computed from alignments with the distmat program in EMBOSS (http://bioweb2.pasteur.fr/docs/EMBOSS/embossdata.html) by using the Tamura distance correction. This method uses transition and transversion rates and takes into account the deviation of GC content from the expected value of 50%. Gap and ambiguous positions were ignored. Final values were then converted to similarity matrices by subtracting each value from 100.

## Results

### Screening of Persons with Respiratory Tract Infections

Persons enrolled in several cohorts of children and adults with respiratory infections (Table) were screened for picornavirus by culture isolation on HE cell lines, real-time PCR specific for HRV-A and HRV-B (*19*), or by a panenterhino real-time PCR designed to theoretically detect all rhinoviruses and enteroviruses with publicly available sequences. Of 1,592 respiratory samples tested by real-time PCR, 248 were virus positive (Table). The 5′-UTR sequences were obtained for 77 real-time PCR or culture-positive samples and VP1 and 3CD sequences for 48 of these (Table; Technical Appendix 1 Table 2). In parallel, the 3CD sequences were identified for all reference serotypes. The results of this screening are summarized in Technical Appendix 1 Table 2, and all sequences are available from GenBank (accession nos. EU840726–EU840988).

On the basis of these results, respiratory infections caused by HRV-B might be less frequent than those caused by HRV-A, and HRV-A infections are distributed among the whole library of reference serotypes. A specific real-time PCR used to detect enteroviruses in respiratory specimens from some of the cohorts studied indicated that these viruses are rare in children (2.5% vs. 6.3% for HRV) and even rarer or absent in adults (0% vs. 24% for HRV) (*28*).

### Phylogeny and Molecular Epidemiology of 5′-UTR

To include all 99 HRV reference strains and new divergent rhinoviruses described recently by Lee et al. (*13*), we reconstructed a phylogenetic tree (Technical Appendix 2 Figure 1, panel A) on the basis of a sequence of 280 nt in the 5′-UTR. This sequence provided a correct clustering of HRV-A, HRV-B, and HEV strains according to the accepted whole-genome phylogeny (Technical Appendix 2 Figure 1, panel D) (*15*) but did not resolve appropriately the phylogeny of the 4 HEV species and the HRV-A and HRV-C viruses. The condensed tree version (Figure 1, panel A) enabled us to identify 2 groups phylogenetically distant from all previously known HRVs and HEVs. The first group, referred to as HRV-C′, contained some of our clinical samples and rhinoviruses sequenced by Lee et al. (*13*). The second group was a new clade and was named EV-104. This clade included 8 clinical samples collected in different regions of Switzerland without direct epidemiologic links (Technical Appendix 1 Table 2).

### Identification of HRV-C Viruses by Sequencing of HRV Viruses with Divergent 5′-UTRs

Characterization of HRVs newly identified during 2006–2008 showed that they all belong to the same HRV-C species (*9*–*16*). Recently, Lee et al. (*13*) identified another cluster of viruses (HRV-C′; Figure 1, panel A) and suggested that this group was phylogenetically distinct from all other HRVs on the basis of analysis of their 5′-UTR sequences. To define the phylogeny, we adapted a previously described method (*23*) to complete the genome sequence directly from our clinical strains (CL-Fnp5 and CL-QJ274218) that showed a similar divergent 5′-UTR (Technical Appendix 2 Figure 1, panel A). A condensed version (Figure 1, panel B) of the phylogenetic tree based on VP1 sequences (Technical Appendix 2 Figure 1, panel B) indicated that CL-Fnp5 clustered with the new HRV-C clade, a finding further confirmed by CL-QJ 274218 partial sequences. This finding supports the view that new HRVs variants described since 2006 (*9*–*16*) all belong to the same lineage.

### New Divergent Lineage of HEV Species C

**Figure 2:**
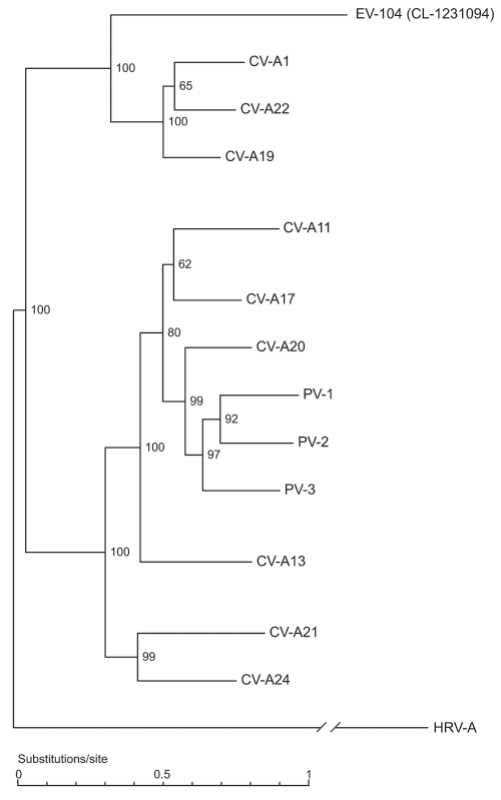
Full genome phylogenetic tree of enterovirus 104 (EV-104), representative strain CL-1231094, and members of the human enterovirus C (HEV-C) species. Human rhinovirus A (HRV-A) (GenBank accession no. DQ473509) was used as outgroup. Coxsackievirus A1 (CV-A1) (AF499635), CV-A21 (AF546702), CV-A20 (AF499642), CV-A17 (AF499639), CV-A13 (AF499637), CV-A11 (AF499636), CV-A19 (AF499641), CV-A22 (AF499643), CV-A24 (D90457), poliovirus 1 (PV-1) (V01148), PV-2 (X00595), and PV-3 (X00925) sequences were obtained from GenBank.

As shown in Figure 1, panel A, the panenterhino real-time PCR enabled detection of a new HEV strain phylogenetically distinct from all previously known HEV species and associated with respiratory diseases. Enterovirus-specific real-time PCRs or reference VP1 primer sets routinely used to type enteroviruses (primers 222 and 224 and nested primers AN88 and 89) (*29**,**30*) did not amplify this new genotype. We could not grow this virus on HeLa and HE cell lines. Consequently, we applied the method described above to complete the genome sequence directly from the CL-1231094 (EU840733) clinical specimen. VP1 and full-length genome sequences showed that, albeit divergent at the 5′-UTR level, this new variant belonged to the HEV-C species (Figure 1, panels B, C). Full-length genome phylogenetic tree (Figure 2) and VP1 protein identity plots (Technical Appendix 2 Figure 2) with all members of the HEV-C species indicated that this virus represents a new HEV-C genotype that shares 68%, 66%, and 63% nucleotide and 77%, 75%, and 68% amino acid sequence identity, respectively, with coxsackieviruses A19 (CV-A19), A22, and A1, the closest serotypes. This new virus was named EV-104 (www.picornastudygroup.com/types/enterovirus_genus.htm).

Specific primers (Ent_P1.29/P2.13 and Ent_P3.30/P3.32; Technical Appendix 1 Table 1C) were then designed to amplify the VP1 and 3D regions of the 7 other samples of this cluster collected from children with acute respiratory tract infections and otitis media. VP1 nucleotide homology among these strains was 94%–98%, except for 1 distantly related sample (74%–76%), which may represent an additional genotype. Additional sequencing is ongoing to verify this assumption.

At the 5′-UTR level, the strain described by Lee et al. (*13*) and EV-104 diverged from other members of HRV-C and HEV-C species, respectively. Thus, the 5′-UTR–based phylogeny was inconsistent with that based on VP1 sequences and suggested possible recombination events (Figure 1, panels A, B). Because the 5′-UTR is the target of most molecular diagnostic assays, this sequence divergence needs to be taken into account in future studies.

### Recombination Events between 5′-UTR, VP1, and 3CD Genome Regions

Other studies have provided sequences of clinical strains, but genetic characterization was often limited to 1 genomic region. Our goal was to sequence 3 genomic regions for each analyzed strain to determine definitively whether recombination events could represent a driving force for the evolution of rhinoviruses in their natural environment. Although recombination events have been suggested for reference serotypes, they have never been shown for circulating clinical strains (*18*,*31**,**32*). In contrast, recombination is well established as a driving force of enterovirus evolution. Thus, we completed the 5′-UTR, VP1, and 3CD sequences of 43 clinical strains by using a pool of adapted and degenerated primers (Technical Appendix 1 Table 1A).

**Figure 3:**
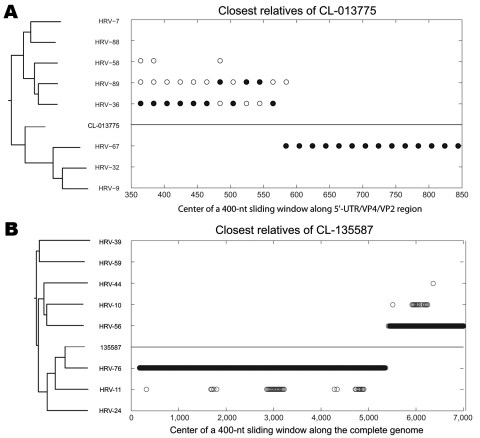
Nearest-neighbor relatedness of rhinovirus CL-013775 (and CL-073908) along the 5′ untranslated region/VP4/VP2 region (A), and nearest-neighbor relatedness of rhinovirus CL-135587 along the complete genome (B), identified by bootscanning. At each position of a sliding window, the solid circles indicate the closest relative within a defined threshold of the phylogenetic distance to CL-013775 (A) and CL-135587 (B). Both panels show phylogenetic trees of analyzed serotypes over the entire scanned region. Human rhinovirus 7 (HRV-7), -9, -10, -11, -24, -32 (accession nos. EU096019, AF343584), -36, -39, -44, -56, -58 (EU096045, AY040236), -59, -67 (EU096054, AF343603, and DQ473505), -76, -88, and -89 sequences were obtained from GenBank (see Technical Appendix 2 Figure 1, for full-length genome accession numbers).

Independent phylogenetic trees (Technical Appendix 2) and similarity matrices were constructed for the 3 genomic regions. Since the last common ancestor and as depicted on the distance matrices and highlighted by boxplots of maximum-likelihood branch length distributions (Technical Appendix 2 Figure 3), there are more mutations fixed in the VP1 region than in the 3CD region, and more in the 3CD region than in 5′-UTR, which is indicative of a variable rate of evolution in these regions. Accordingly, VP1 sequences enabled genotyping of all but 3 clinical strains analyzed (Technical Appendix 2, Figure 1, panel B). These strains may represent rhinovirus genotypes only distantly related to predefined reference serotypes. In contrast, genotyping based on 3CD and 5′-UTR was less accurate, as expected. These results confirmed that molecular typing of rhinoviruses, similarly to other picornaviruses, must use capsid sequences.

Phylogeny of the 5′-UTR, VP1, and 3CD of reference serotypes showed many incongruities caused by insufficient tree resolution or recombinant viruses as previously proposed (*18**,**31*). As an example, 2 VP1 clusters including HRV-85/HRV-40 and HRV-18/HRV-50/HRV-34 (Technical Appendix 2 Figure 1, panel B) were reorganized as HRV-85/HRV-18/HRV-40 and HRV-50/HRV-34, respectively, on 3CD (Technical Appendix 2 Figure 1, panel C). The differential cosegregations between these virus strains suggested recombination events. When available, full-length genome sequence bootscanning applied to all serotypes will give an estimate of the number of reference strains with mosaic genomes.

Similarly, the noncoding region, VP1, and 3CD trees showed major phylogenetic incongruities for 3 clinical isolates (Technical Appendix 2 Figure 1). Two of these isolates (CL-013775 and CL-073908) were typed as HRV-67 on the basis of VP1 sequence and were closest to this serotype in 3CD, whereas the 5′-UTR cosegregated with HRV-36 (see 5′-UTR recombinant; Technical Appendix 2 Figure 1, panels A–C). These viruses were isolated by cell culture from 2 epidemiologically linked cases and thus represented transmission of the same virus. To confirm the recombination, we completed the sequencing by obtaining the 5′-UTR, VP4, and VP2 sequences (EU840918 and EU840930) and compared them with HRV-36, HRV-67, and other closely related serotypes. Bootscanning analysis (Figure 3, panel A) enabled mapping of the recombination site within the 5′-UTR, just before the polyprotein start codon. Sequence alignment mapped recombination breakpoints more precisely between positions 524 and 553 with reference to HRV-2 (X02316).

The other incongruent isolate (CL-135587) was typed as HRV-76 on the basis of VP1 sequence and was closest to this serotype in the 5′-UTR, but 3CD cosegregates with HRV-56 (3C recombinant; Technical Appendix 2 Figure 1, panels B, C). Similarly, we completed the full-length sequence of this isolate (EU840726) and HRV-56 (EU840727). The same approach enabled mapping of the recombination site at the N terminus of protein 3C between positions 1511 and 1523 with reference to HRV-2 (Figure 3, panel B). These results demonstrate that recombination occurs among clinical rhinoviruses. In our analysis of 40 rhinovirus-positive samples collected over 9 years (3 additional samples were duplicates of 2 different viruses; Technical Appendix 1 Table 2) for 3 genomic regions, 2 of the analyzed viruses appeared to be recombinants. The 2 documented recombinations occurred in members of the HRV-A species. The design of this study and technical issues (e.g., inability to sequence low viral loads) limited the ability to calculate a recombination rate, particularly for HRV-B and HRV-C.

## Discussion

Our genomic analysis of picornaviruses associated with upper or lower respiratory diseases in adults and children indicates that rhinoviruses circulating in the community are widely diverse. The large number of circulating genotypes supports the view that rhinoviruses do not circulate by waves or outbreaks of a given dominant genotype, which might explain the high frequency of reinfection during short periods. As expected, the observed variability is higher for surface capsid proteins, the targets of most immune pressure, and this region remains the only accurate one for genotyping and defining phylogeny. Technical constraints such as the limited amount of clinical specimens, the use of different screening methods, and the need to sequence an unknown target of extreme variability might have limited the representativeness of our sequence collection. Therefore, our study should not be considered as an exhaustive epidemiologic analysis of rhinoviruses and enteroviruses associated with respiratory diseases.

By using a systematic approach, we have identified a new enterovirus genotype (EV-104) that has a divergent 5′-UTR. Undetectable by conventional methods, EV-104 could be detected by using a more generic real-time PCR assay designed to match all known available rhinovirus and enterovirus sequences. Such diagnostic tools have and will lead to constant discovery of new picornavirus genotypes (*9*–*14*,*16*,*33*–*36*). These genotypes may represent viruses, in most instances, that have remained undetected because of insensitive cell cultures or overly restrictive molecular tools. In addition, enterovirus genotypes causing respiratory infections, such as EV-68 and CV-A21, might be underrepresented because enteroviruses are usually searched for in fecal specimens (*37*).

EV-104 belongs to the HEV-C species: CV-A19, CV-A22, and CV-A1 are its closest serotypes. These HEV-C subgroup viruses are genetically distinct from all other serotypes of the species. These viruses show no evidence of recombination with other HEV-C strains and, similar to EV-104, do not grow in cell culture (*29*). On the basis of our epidemiologic data, we conclude that EV-104 was found in 8 children from different regions of Switzerland who had respiratory illnesses such as acute otitis media or pneumonia. Future studies using adapted detection tools will provide more information on the range of this virus. On the basis of its genomic features and similarities with coxsackieviruses and poliovirus, EV-104 could theoretically infect the central nervous system (*2*,*38*). Detection of new subtypes of picornaviruses indicates that viruses with new phenotypic traits could emerge, and conclusions on tropism of new strains should be substantiated by extensive experimental or clinical investigations (*39*).

By completing the sequence of a seemingly divergent rhinovirus (*13*), we assigned this virus to the new HRV-C species, thus limiting currently to 3 the number of HRV species. For the sake of simplicity, we propose to consider this virus as a member of the HRV-C clade.

Finally, we demonstrated that rhinovirus evolves by recombination in its natural host. Known to be a driving force of enterovirus evolution, rhinovirus recombination among clinical strains has never been observed. Two clinical isolates of 40 viruses analyzed resulted from recombination events and their breakpoints were identified within the 5′-UTR sequence and the N terminus of protein 3C, respectively. These findings are consistent with the fact that recombination breakpoints in picornaviruses are restricted to nonstructural regions of the genome or between the 5′-UTR and the capsid-encoding region (*40*). Our observations provide new insight on the diversity and ability of rhinovirus to evolve in its natural host. The fact that only 2 of 40 analyzed viruses over a 9-year period were recombinants is suggestive of a lower recombination frequency in rhinoviruses than in other picornaviruses (*32**,**40*) and might be related, but not exclusively, to the short duration of rhinovirus infection (*18**,**31**,**32*). Recombination events occurred between HRV-A genotypes, but whether they can occur in species B and C remains unknown. Interspecies recombination is rare in picornaviruses and is mainly the result of in vitro experiments. For rhinoviruses, the different location of *cre* elements in each species might be an additional limiting constraint (*17*).

In summary, we have highlighted the large genomic diversity of the most frequent human respiratory viral infection. Our phylogenetic analysis has characterized circulating strains relative to reference strains and has identified a previously unknown enterovirus genotype. We have shown that recombination also contributes to rhinovirus evolution in its natural environment.

## Supplementary Material

Technical Appendix 1 New Respiratory Enterovirus and Recombinant Rhinoviruses among Circulating Picornaviruses

Technical Appendix 2 Untranslated region (UTR) (A), capsid protein VP1 (B), protease precursor 3CD (C)
