# Supplementary material for: New Respiratory Enterovirus and Recombinant Rhinoviruses among Circulating Picornaviruses
Source: Emerg Infect Dis. 2009 May;15(5):719–26. doi: 10.3201/eid1505.081286 (PMC2687021; doi:10.3201/eid1505.081286)
Supplement: Technical Appendix 1 — New Respiratory Enterovirus and Recombinant Rhinoviruses among Circulating Picornaviruses [file 08-1286_Techapp1-s1.pdf]

Technical Appendix 1

Technical Appendix 1 Table 1A. Primers used to amplify and sequence the 5′ untranslated region, capsid protein VP1, and protease precursor 3CD regions of clinical strains and reference serotypes of human rhinovirus (HRV)

ID	Sequence (5′ → 3′)	Suited for	Start*	End*
4	CTACTTTGGGTGTCCG	Most HRV	547	532
6	CGGACACCCAAAGTAG	Most HRV	532	547
10	GTACWCTRKTAYTMYGGTAMYYTTGTACGCC	Most HRV	49	80
11	GCACTTCTGTTTCCCC	Most HRV	164	179
14	ATTCAGGGGCCGGAGGA	Most HRV	450	434
19	CGTTAYCCGCAAGRYGCCTAC	Most HRV	223	244
22	AGCCTGCGTGGCKGCC	Most HRV	350	366
23	GAAACACGGACACCCAAAGTAGT	Most HRV	554	531
P1.100	TGTTTGTACATATCGTGTTTCAATC	HRV-61	2486	2461
P1.101	TCAGTGCTAGCCATTTTAGG	HRV-76	2109	2129
P1.102	TTTGTGGTCATCAGTTACTATCC	HRV-76	2990	2967
P1.103	AAGTTCTGAATGAGGTCCTTG	HRV-73	2337	2358
P1.104	TCATTTCTTTTTGCAGGTAGAG	HRV-73	2805	2783
P1.105	CCATTAGTCTTGAGAGCAGTGC	HRV-73	1817	1839
P1.106	GCAGCATCCAGTGCAGG	HRV-73	2422	2405
P1.107	CCTGGTATTGGAGAGCCTGC	HRV-73	2069	2086
P1.108	TCTCTGCTATCTGGTACTGGAGC	HRV-73	2046	2023
P1.109	GTATCTCTAGTTGTCCCATGGGT	HRV-29	2087	2110
P1.110	CATTTCAAATTTACGTCTAATTTGAG	HRV-29	2651	2625
P1.111	CACATGCAGCCCTACC	HRV-27	2157	2141
P1.112	ACCATCATAATGCATATATGTGG	HRV-27	2515	2492
P1.113	GTTGGCTTGCAATCCAC	HRV-94	1990	2007
P1.114	GTGATTTGCCATGATTGG	HRV-94	2622	2604
P1.115	AACCCAGTGGAAGAGTTTGTGGAG	QPM	2127	2151
P1.116	GTTAGTGATACTTGTGATGTCTTGTCTGTAC	QPM	2949	2918
P1.117	CACTCATTGGACAGGCAGCTTTGC	HRV-58	1918	1942
P1.118	GGAGACGGGGTGTTCAAGACTACA	HRV-17/-70	1873	1897
P1.119	CTGAGCCCTGTGCTCCTGTTGCTAC	HRV-17/-70	3356	3331
P1.12	AGTGCATCTGGTAATTTCCA	Most HRV	1055	1075
P1.120	GCATATACACCACCTGGAGTACCAAAACC	HRV-68	1994	2023
P1.121	GGGTAATATTCTGATTCCTGAATTTCATACC	HRV-68	3421	3390
P1.122	GGTTGGTGCCATGGGTCAGTGC	HRV-57	2094	2116
P1.123	CCCTCACCTATTAACAAATTATACTGTATG	HRV-57	3457	3427
P1.124	GGATACATAACATGCTGGTATCAAACCAG	HRV-49	2156	2185
P1.125	GCAGTCCACTATATCAATGATAGTGCC	HRV-22	2077	2104
P1.126	GGATAGTACTCACTCTCTTGAATTTCATACC	HRV-22	3421	3390
P1.127	GTGCCAAGTTGATACGATCATCCC	HRV-19	1740	1764
P1.128	GCCAAGTGGATTCTATGATACCTGTAAAC	HRV-46	1751	1780
P1.129	CCAGCTATTACACCCATTGGACAGG	HRV-71	1908	1933
P1.130	ACACCCTCCTCTCCATGCACTAA	HRV-71	2554	2531
P1.131	GAAATGTGCCAAGTAGACACAATTATACC	HRV-67	1733	1762
P1.132	CCTGACCTTCCAAGGAAGCTTTC	HRV-67	2536	2513
P1.133	GTCCCATGTGTGCAGAGTTTTCTGGAG	HRV-68	1557	1584
P1.148	CATCTCTAGTCTGTGATGTTTGCAC	HRV-66	2501	2477
P1.149	GATATATGGATACACCCTGACCTACC	HRV-66	2551	2525
P1.18	TGTKCGRTAWATGATTARATC	Most HRV	3284	3263
P1.2	TGGTGSTGGAARYTRCCWGATGC	Most HRV	1049	1072
P1.24	TAYTCWSWYTCYTGDATYTCRTACCA	Most HRV	3415	3389
P1.26	TYTCYTGDATYTCATACCARTCATG	Most HRV	3408	3383
P1.28	TACTACATGTTTTATGATGGGTATG	HRV-94	2882	2907
P1.43	GACACTTACTCAAAAGCTGG	HRV-12/-78	2142	2158
P1.55	GCTTCAGYTTCATGTTTTGTGG	Most HRV	1941	1963
P1.57	CCWRCACTRACWGCWAATGAAACTGG	Most HRV-B	2419	2445
P1.58	TCATGCTCATTRACYAYYCTRAAAGCYAT	Most HRV-B	3024	2996
P1.6	ATGGCCCAGATTAGAAGAAAG	Most HRV	2621	2642
P1.63	CCYATGTATGCAGARTTYTCYGG	Most HRV-A	1559	1582
P1.65	TTYCTYCTAATYTGRGCCATYTC	Most HRV-A	2642	2618
P1.72	GGWCAGTTYHTGACAACAGATGA	Most HRV-A	1631	1654

P1.82	GATGAGATGAGTGTTGAAAGTTTCTTAGG	HRV-24/-11	2501	2527
P1.95	GGCAATGTCAATATGTACACAG	HRV-16	1802	1824
P1.96	GCCTGCTACACTTGGTACC	HRV-16	2720	2701
P1.97	CAAGAGCAAAACCGATGG	HRV-59	1585	1603
P1.98	ATGTGTATCAGTGTCTCTGGC	HRV-59	2304	2290
P1.99	TGCTTTACCTTGGTATCATCCC	HRV-61	1669	1691
P2.19	TGTRTACAYCTRAGTATVCCACCACA	HRV-B	3543	3517
P2.42	CTACAAACCCATTGCCAAAAGCTTC	HRV-11/-24	3642	3617
P3.1	CARGGNCCWTAYTCNGG	Most HRV	5066	5082
P3.28	TGGAATTCATGTGGGTGGTAAT	HRV-3 /-37	5605	5627
P3.3	TCAWARTTWGWRTAATCAAANGCCAT	Most HRV	6396	6371
P3.50	GTACTGCGTGCAGTAGTTACCCAAGG	HRV-3/-37	5112	5138
P3.56	GACATCTGGATCACATCCCACTGC	HRV-93/-27	6329	6305
P3.59	AGCAARTCATAAGGAGGKAYATACA	HRV-A	7036	7011
P3.63	CAG AGA ATG AGG ATG ATT ACC CAG ATT G	HRV-11/-24	5403	5431
P3.69	TYAAWCCATATTTMWYACCTTCCWTDGC	HRV-A	6735	6707
P3.7	ACNAGTGCWGGNTWYCCNTAT	Most HRV	6020	6040
P3.77	CATGTGGGTGGTAATGGC	HRV-75	5612	5630
P3.78	GATCATTCATCTCACATTCAGGA	HRV-75	5878	5855
P3.79	GAAATCAAACAGCAAGAATGC	HRV-54	5517	5538
P3.8	GGNTYCTTDGTCCATC	Most HRV	6898	6882
P3.80	AGTTCATCTTTAAGGAATGTGACC	HRV-54	6166	6142
P3.81	GGHAAATTYACAGGBYTAGGHATHTATGA	HRV-A	5198	5227
P3.82	AAAACTCCACCACAATACCC	HRV-61	5584	5564
P3.83	GGNATWCATGTNGGWGGYAATGG	HRV-A	5606	5629
P3.84	CANCCWGATGGCAYWCCWCC	HRV-A	6547	6527
P3.85	CCAATTTGGGCTARATAASYCATGTC	HRV-8/-45/-95	6692	6666
P3.86	TAGATAAATATGGTGTGGATCTGCC	HRV-20	6115	6140
P3.87	GAAAAKATNACATCATCWCCATAWGC	HRV-A	6676	6651
P3.88	CAAGGRCCWGARGAAGARTTTGG	HRV	5129	5152
P3.89	GGATCWTTCTCTGAWAGYACWGCTGG	HRV-8/-45/-95	5824	5798
P3.90	GGTAACATTCCTTAAAGATGAACTCAG	HRV-61	6142	6169
P3.91	CGTGTTCCTGCATTTGTGAGGGC	HRV-61	6918	6895
P3.92	CACAGGGATAGGACATGTTCTTGC	HRV-68	6931	6906
P3.93	GAATTCATGTGGGGGGCAATG	HRV-67	5607	5628
P3.94	CTACTGGCCTCTATAACTCTAGTCTTGCC	HRV-67	6220	6191
P3.95	GGGTAAAACTAGAGTCATTGAGGCTAG	HRV-49	6190	6217
P3.96	GTAAATCAACCCCATACTGATCCAGC	QPM	5944	5918
P3.97	GCATGGAAGGTACTGAAGAGATTGCC	QPM	6086	6060
P3.98	CTAAGCTCATCTTTTAAGAAAGTAACCATG	HRV-58	6169	6139

*Positions relative to HRV-2.

Table 1B. Primers used to amplify and sequence the genomes of human rhinovirus C′ strains

ID	Sequence (5′ → 3′)	Start*	End*
FR26RV-N	GCCGGAGCTCTGCAGATATNNNNNN	–	–-
FR20RV	GCCGGAGCTCTGCAGATAT	–	–
32	GCTCAGCAGTACCCCACTGTAGA	318	341
33	GCCTGCGTGGTGCCC	351	366
P1.153	CCATACATCAACTGTGTACCCATGGAC	1352	1379
P1.154	CACAACAACTTCAGTCTGGTGATTGTACC	1469	1498
P1.155	CAAGACTCAATTCAGCCCACAATG	2514	2490
P1.156	GAGCTTGTTCTTGGAAGCTAATATCCC	2532	2505
P1.157	GAGCACTTGAAGATGTGCAGGG	2939	2961
P1.158	GCACGGTGTCCTCGCG	2841	2857
P1.159	GTAGTGCTCTTCTTTCAGGACTTGTGTGTC	3023	2993
P1.160	TRGCMTACACMCCHCCAGG	1920	1939
P1.161	GCATGHTRCGTGAYACACCMATGAT	2190	2215
P1.162	CTTCAGAGCACTTGAAGATGTGCAG	2995	3020
P1.163	AACTATGGTATATCTGCCACTAATGATATGGG	2810	2842
P1.164	CGTCTTGGCATATGGAGAATGG	835	857
P1.165	CTAGGTGCGCGAGGACACC	3064	3046
P1.170	CCTAGATGCCACTTTCGTAGATAAACCATC	874	904
P1.171	CTGTACCCTGCACATCTTCAAGTGC	2858	2833
P2.68	TTRCCWCCACAGTCTCCWGG	3376	3356
P2.69	GTRTGAACAAABAKRTCACTRGGTCC	3079	3053
P2.70	TGTTGTCCWYTTCTATGTGG	4607	4627
P2.71	CCWYTTCTATGTGGYARGC	4613	4632
P2.72	GAAGGGGACATATATTCTTTACCGCCC	4214	4241
P2.73	CCAATCCCAAACACTTTGACGGG	4239	4262
P2.74	GCTGGTGGTGAGGACCATGTG	3413	3434
P2.75	CCGCAAGAGGGCCTCAGG	4466	4448
P2.76	CCCTCCTGTGTGGCAAGGC	4614	4633
P2.77	GGTATCCTCTTACAATGTTTCCAGCAGC	3937	3909
P2.78	GTGGCATTTACCGATCTCAGGC	3431	3453
P3.107	TCCTTWGTCCATCTRATTGAYTCA	6805	6781
P3.108	TTTRAGRAAGGTVACAKTVTCCCA	6713	6689
P3.109	TGGGTCATTSAKACTKGCTGCTTC	6146	6122
P3.110	CCTTASRGCYATTTTTCARGGTTTGGG	4735	4762
P3.111	GACCCAACATCAGCAAAGAACATG	5398	5374
P3.112	CCTGGTCAATGTGGGGGTGTC	5474	5495
P3.113	CCACTTTAGGGGTCAAGAAGAGGG	5435	5459
P3.114	TAYGGCTCYTCACCATTGTGCCA	6874	6851
P3.115	GGTCTTGTGTATTYTTGGGRTC	6825	6803

*Positions relative to NAT045.

Table 1C. Primers used to amplify and sequence the genomes of clinical strains of enterovirus 104

ID	Sequence (5′ → 3′)	Start*	End*
34	CGGCCTGCCCATACCC	370	386
35	CGGTATGGGACGCTCACTTC	388	406
Ent_P1.27	TTCTAGCTTGCGGCGC	2816	2800
Ent_P1.28	GGTGAGATTCTVAAYTACTACACHCACTGG	2039	2069
Ent_P1.29	ATGYTNGGYACYCACWTRATATGGGA	2183	2209
Ent_P1.30	GATTCTCAACTACTACACCCACTGG	2044	2068
Ent_P1.31	CTTGGTACCCACTTAATATGGGA	2186	2209
Ent_P2.10	GARGCDTGYAANGCWGCYAARGG	4115	4138
Ent_P2.11	TCAATRCGGTGTTTGSTCTTGAACTG	4450	4424
Ent_P2.12	CATACTGGTTCAATRCGGTGTTTG	4459	4435
Ent_P2.13	AGCACTRAAMCCMGCACCAAA	3854	3833
Ent_P2.14	TCTTTRAGCCATTGCCATGG	4045	4025
Ent_P2.15	GCACTAAACCCAGCACCAAA	3852	3833
Ent_P2.16	TCTTTAAGCCATTGCCATGG	4045	4025
Ent_P2.8	GARGADGARGCMATGGARCARGG	3779	3799
Ent_P2.9	TGGHTRAAGAARTTYACNGARGC	4097	4120
Ent_P3.25	TTYGAYTAYGCWGTNGCHATGGC	5414	5437
Ent_P3.26	TAYGCAGTNGCHATGGCHAARARRAACAT	5420	5449
Ent_P3.27	CCAACATGYATSCCRATNACYTTVCCAGT	5893	5864

Ent_P3.28	AANCCRTGDGAHCCRTTMCCDCCAAC	5914	5888
Ent_P3.29	GARGAYGCHATGTAYGGNACNGATGG	6271	6245
Ent_P3.30	ATGGTKYTDGARAARATTGGWTTTGG	6704	6730
Ent_P3.31	GGATCYTTDGTCCAYCTRATWGATTC	7174	7148
Ent_P3.32	AATNTCYTTCATKGGCATYACTGG	7145	7121
Ent_P3.33	CGCAACAGATACACCCTTGATGAGC	4979	5300
Ent_P3.34	GCAAGGCAATCCAATTGATGGAC	4947	4970
Ent_P3.35	TGGACCTTGGACCTTGGC	5411	5395
Ent_P3.36	CCAATGATGCGGTACTTGCCG	5694	5715
Ent_P3.37	TACCCTTTACGTGGCAATGGG	6305	6325
P1.150	GAGTTTATGATGGCCAGCAACG	1307	1329
P1.151	CGTCACTTACGAGCGGGCC	1348	1367
P1.152	GCAAATGAATTTTTGGCGGG	1444	1424
P2.65	GGTGCCCACGCCCACC	3249	5138
P2.66	GCCGCAGCTGACATACGC	3326	5627
P2.67	CATGCTAGCGTTTATCAGTGCATG	2365	2389

*Positions relative to coxsackievirus A19 or enterovirus 104.

Table 1D. Primers used to amplify and sequence the genomes of recombinant and parent human rhinovirus (HRV)

ID	Sequence (5′ → 3′)	Virus	Start*	End*
16	AGCCTCATCTGCCAGGTCTA	HRV	322	302
28	AAACTGGATCYAGGTTGTTCCCACCT	HRV	4	30
P1.45	GTTGTTAAGAATTGACCAGATCC	HRV-12/-78	1648	1625
P1.49	AAGCAAATTCACTGATCCAG	HRV 78	760	780
P1.55	GCTTCAGYTTCATGTTTTGTGG	HRV-A	1941	1963
P1.72	GGWCAGTTYHTGACAACAGATGA	HRV-A	1631	1654
P1.80	CAAGTGTAAATTGGTATCCCGTGCC	HRV-11/-24	2291	2266
P2.1	GARCYWGGWGAYTGYGWGG	HRV	3464	3484
P2.2	GGRTTYTGSWTNANRTCATCCAT	HRV	4417	4395
P2.3	CCNCCWGAYCCNAARYAYTTTGATGG	HRV	4322	4347
P3.2	CKNATRTCYYTRAAYTTYTCATTTCT	HRV	5391	5366

*Positions relative to HRV-2.

Table 2. Sequence characterization of virus samples included in the study*

Type of study	Sample name	GenBank accession nos.	Origin	Genotyping			Species
				5′ untranslated region	Capsid protein VP1	Protease precursor 3CD	
Routine isolation (1999–2008)	CL-080070	EU840932/ EU840886/ EU840764	Isolated by cell culture from NPS	HRV-59 (NS)	HRV-59	HRV-59	HRV-A
	CL-155838	EU840951/ EU840895/ EU840773	Isolated by cell culture from BAL	Untypeable (NS)	HRV-15	HRV-15	HRV-A
	CL-elLCT220052	EU840973/ EU840908/ EU840785	Isolated by cell culture from NPS	HRV-12	HRV-12	HRV-12	HRV-A
	CL-240132	EU840956/ EU840900/ EU840777	NA	Untypeable (NS)	HRV-29/-44	HRV-29/-44	HRV-A
	CL-043679	EU840926/ EU840880/ EU840758	Isolated by cell culture from BAL	HRV-54 (NS)	HRV-54	HRV-54	HRV-A
	CL-274951	EU840958/ EU840901/ EU840779	Isolated by cell culture from BAL	Untypeable (NS)	HRV-29/-44	HRV-29/-44	HRV-A
	CL-145608	EU840950/ EU840894/ EU840772	Isolated by cell culture from BAL	Untypeable (NS)	HRV-47	HRV-47	HRV-A
	CL-135587†	EU840726	Isolated by cell culture from BAL	HRV-76 (NS)	HRV-76	HRV-56	HRV-A

	CL-030118	EU840922/ EU840876/ EU840754	Isolated by cell culture from PS	HRV-16 (NS)	HRV-16	HRV-16	HRV-A
	CL-100091	EU840933/ EU840887/ EU840765	Isolated by cell culture from BAL	HRV-61 (NS)	HRV-61	HRV-61	HRV-A
	CL-110090	EU840934/ EU840888/ EU840766	Isolated by cell culture from NPS	HRV-61 (NS)	HRV-61	HRV-61	HRV-A
	CL-030103	EU840921/ EU840875/ EU840753	Isolated by cell culture from BAL	HRV-34 (NS)	HRV-34	HRV-34	HRV-A
	CL-023751	EU840919/ EU840873/ EU840751	Isolated by cell culture from NPS	HRV-7	HRV-7	HRV-7	HRV-A
	CL-310106	EU840961/ EU840904/ EU840782	Isolated by cell culture from NPS	Untypeable (NS)	HRV-1B	HRV-1B	HRV-A
	CL-030119	EU840923/ EU840877/ EU840782	Isolated by cell culture from NPS	Untypeable (NS)	HRV-1A	HRV-1A	HRV-A
	CL-040055	EU840925/ EU840879/ EU840757	NA	HRV-75 (NS)	HRV-75	HRV-75	HRV-A
	CL-064493	EU840928/ EU840882/ EU840760	NA	HRV-34 (NS)	HRV-34	HRV-34	HRV-A
	CL-080053	EU840931/ EU840885/ EU840763	Isolated by cell culture from PS	HRV-46	HRV-46	HRV-46	HRV-A
	CL-200087	EU840954/ EU840897/ EU840775	Isolated by cell culture from NPS	HRV-71	HRV-71	HRV-71	HRV-A
	CL-039885	EU840924/ EU840878/ EU840756	Isolated by cell culture from NPS	HRV-19	HRV-19	HRV-20/-19	HRV-A
	CL-144349‡	EU840948/ EU840892/ EU840770	Isolated by cell culture from NPS	HRV-94	HRV-94	HRV-94	HRV-A
	CL-144350‡	EU840949/ EU840893/ EU840771	Isolated by cell culture from NPS	HRV-94	HRV-94	HRV-94	HRV-A
	CL-246706‡	EU840957/ EU840899/ EU840778	Isolated by cell culture from PS	HRV-94	HRV-94	HRV-94	HRV-A
	CL-290046	EU840960/ EU840903/ EU840781	Isolated by cell culture from PS	HRV-22	HRV-22	HRV-22	HRV-A
	CL-070102	EU840929/ EU840883/ EU840761	Isolated by cell culture from BAL	HRV-78	HRV-78	HRV-78	HRV-A
	CL-279529	EU840959/ EU840902/ EU840780	Isolated by cell culture from NPS	HRV-24	HRV-24	HRV-24	HRV-A
	CL-170122	EU840953/ EU840896/ EU840774	Isolated by cell culture from PS	HRV-9	HRV-9	HRV-9	HRV-A
	CL-210068	EU840955/ EU840898/ EU840776	Isolated by cell culture from BAL	HRV-57	HRV-57	HRV-57	HRV-A
	CL-029646	EU840920/ EU840874/ EU840752	Isolated by cell culture from PS	HRV-73	HRV-73	HRV-73	HRV-A
	CL-013775‡	EU840918/ EU840872/ EU840750	Isolated by cell culture from PS	HRV-36	HRV-67	HRV-67	HRV-A

	CL-073908‡	EU840930/ EU840884/ EU840762	Isolated by cell culture from PS	HRV-36	HRV-67	HRV-67	HRV-A
	CL-170085	EU840952	BAL	New	NA	NA	HRV-C
Infection in newborns (1999–2005)	CL-DY311099	EU840972/ EU840907/ EU840784	NPS	New (NS)	New	New	HRV-C
	CL-Bern226	EU840963	NPS	HRV-45	NA	NA	HRV-A
	CL-Bern230	EU840964/ EU840906	NPS	New	New	NA	HRV-C
Hospitalized patients (2003–2006)	CL-Fnp5§	EU840728	NPS	New	New	NA	HRV-C'
	CL-Fnp507	EU840977/ EU840910/ EU840786	NPS	HRV-68	HRV-68	HRV-28/-68	HRV-A
	CL-Lba198	EU840980/ EU840911/ EU840788	BAL	HRV-58	HRV-58	HRV-58	HRV-A
	CL-Lba236	EU840982/ EU840912/ EU840789	BAL	Untypeable	HRV-70	HRV-70	HRV-B
	CL-Lba503	EU840983/ EU840913/ EU840790	BAL	HRV-68	HRV-68	HRV-28/-68	HRV-A
	CL-Lba516	EU840984/ EU840914	BAL	New	New	NA	HRV-C
	CL-Fnp543	EU840978/ EU840787	NPS	New	NA	New	HRV-C
	CL-Fnp129	EU840974	NPS	New	NA	NA	HRV-C'
	CL-Fnp260	EU840975	NPS	Untypeable	NA	NA	HRV-A
	CL-Fnp409	EU840976	NPS	HRV-58	NA	NA	HRV-A
	CL-Lba202	EU840981	BAL	HRV-55	NA	NA	HRV-A
Hospitalized patients (2001–2003)	CL-060043	EU840927/ EU840881/ EU840759	BAL	HRV-27 (NS)	HRV-27	HRV-27	HRV-B
	CL-aaLba1089	EU840962/ EU840905/ EU840783	BAL	HRV-93	HRV-27	HRV-27	HRV-B
	CL-perLCT080057	EU840985/ EU840915/ EU840791	Isolated by cell culture from NPS	HRV-64	HRV-64	HRV-64	HRV-A
	CL-ruLba1009	EU840987/ EU840916/ EU840792	BAL	HRV-24	HRV-24	HRV-24	HRV-A
	CL-vaFnp389	EU840988/ EU840917/ EU840793	NPS	Untypeable	HRV-3	HRV-3	HRV-B
	CL-QJ274218§	EU840729/ EU840730/ EU840730/ EU840732	BAL	New	NA	NA	HRV-C'
Acute respiratory tract infection (2004–2007)	CL-1432930	EU840941/ EU840890/ EU840768	NPS	HRV-49	HRV-49	HRV-49	HRV-A
	CL-1433740	EU840942/ EU840891/ EU840769	NPS	Untypeable	Untypeable	Untypeable	HRV-A
	CL-1433741	EU840943	NPS	HRV-49	NA	NA	HRV-A
	CL-1434143	EU840944	NPS	New	NA	NA	HRV-C
	CL-1434714	EU840945	NPS	HRV-49	NA	NA	HRV-A
	CL-1434715	EU840946	NPS	Untypeable	NA	NA	HRV-A
	CL-1438069	EU840947	NPS	New	NA	NA	HRV-C
	CL-1230139	EU840935	NPS	HRV-66	NA	NA	HRV-A
	CL-1237693	EU840939	NPS	New	NA	NA	HRV-C
	CL-1235044	EU840936	NPS	New	NA	NA	HRV-C
	CL-1236331	EU840937	NPS	New	NA	NA	HRV-C
	CL-1236333	EU840938	NPS	New	NA	NA	HRV-C

	CL-1227499	EU840735	NPS	Untypeable	NA	NA	HEV-C
	CL-1231094†	EU840733	NPS	Untypeable	EV-104	EV-104	HEV-C
	CL-1232386	EU840737/ EU840740/ EU840746	NPS	Untypeable	EV-104	EV-104	HEV-C
	CL-1234691	EU840738/ EU840741/ EU840747	NPS	Untypeable	EV-104	EV-104	HEV-C
	CL-1231100	EU840736/ EU840739/ EU840745	NPS	Untypeable (NS)	EV-104	EV-104	HEV-C
	CL-1243049	EU840742/ EU840748	NPS	NA	EV-104	EV-104	HEV-C
	CL-1248803	EU840743	NPS	NA	EV-104	NA	HEV-C
Acute infection in children (2006–2007)	CL-C22	EU840734/ EU840744/ EU840749	NPS	Untypeable	EV-104	EV-104	HEV-C
	CL-C31	EU840965	NPS	HRV-56	NA	NA	HRV-A
	CL-C33	EU840966	NPS	HRV-66	NA	NA	HRV-A
	CL-C37	EU840967	NPS	HRV-28	NA	NA	HRV-A
	CL-C47	EU840968	NPS	HRV-59	NA	NA	HRV-A
	CL-C51	EU840969	NPS	HRV-59	NA	NA	HRV-A
	CL-C5	EU840970	NPS	HRV-49	NA	NA	HRV-A
	CL-C62	EU840971	NPS	HRV-34	NA	NA	HRV-A

*NPS, nasopharyngeal sample; HRV, human rhinovirus; NS, not shown in phylogenetic tree because of size <280 nt; BAL, bronchoalveolar lavage fluid; NA, not available; PS, pharyngeal sample.
†Complete genome sequenced,
‡Transmission of a unique clinical strain.
§Partial genome sequenced.
