# Supplementary material for: New Respiratory Enterovirus and Recombinant Rhinoviruses among Circulating Picornaviruses
Source: Emerg Infect Dis. 2009 May;15(5):719–26. doi: 10.3201/eid1505.081286 (PMC2687021; doi:10.3201/eid1505.081286)
Supplement: Technical Appendix 2 — Untranslated region (UTR) (A), capsid protein VP1 (B), protease precursor 3CD (C) [file 08-1286_Techapp2-s2.pdf]

Technical Appendix 2

Technical Appendix 2 Figure 1. 5′ untranslated region (UTR) (A), capsid protein VP1 (B), protease precursor 3CD (C), and complete genome cladograms (D) of rhinoviruses and enteroviruses isolated from patients with respiratory tract infections (see Table 1 in main text and online Technical Appendix 1 Table 2, available from www.cdc.gov/EID/content/15/5/719-Techapp1.pdf), of 101 human rhinovirus (HRV) prototype strains of available sequences, and of the new HRV-C species and samples of human enterovirus A (HEV-A), -B, -C, and -D (panels A, B, and D). 5′-UTR and 3C recombinants are indicated in blue and red, respectively. HRV-C′ and EV-104 viruses are indicated in green and orange, respectively, and the VP1 untypeable viruses are indicated in purple (panel B). Strains discussed in the text are indicated by gray boxes. Simian picornavirus 1 (SV-2) (GenBank accession no. AY064708) was used as outgroup. Rhinovirus 5′-UTR sequences (EU126663–763), VP1 sequences (AY355180–281), full-length or 3CD sequences (DQ473485–512 and EF173414–25, CL-EF582384–6, EF186077 for QPM, EF077280 for Nat045; EF077279 for Nat001), and enterovirus full-length sequences (AF499635 for coxsackievirus A1 [CV-A1], AF499641 for CV-A19, V01148 for poliovirus 1A [PV-1], X00595 for PV2, X00925 for PV-3, AF081485 for CV-B2, AF029859 for echovirus 1 [E-1], AY302558 for E-6, DQ201177 for EV-70, AY426531 for EV-68, AY421760 for CV-A2, AY421764 for CV-A6, and AY421769 for CV-A14) were obtained from GenBank.

Technical Appendix 2 Figure 2. Capsid protein VP1 sequence identity over the entire human enterovirus C species. *CV-A15 (AF499638) and **CV-A18 (AF499640) were recently reclassified as strains of coxsackievirus A11 (CV-A11) and CV-A13, respectively. PV, poliovirus; EV, enterovirus.

Technical Appendix 2 Figure 3. A) Boxplots of branch length distributions of human rhinoviruses (HRVs) based on maximum-likelihood trees. Horizontal lines indicate means, boxes indicate interquartile ranges (i.e., starting at the first quartile and ending at the third quartile), error bars (whiskers) indicate data within 1.5× the interquartile range above or below the first or third quartiles, and dots indicate data points (outliers) too distant from the mean. NCR, noncoding region; VP1, viral capsid protein 1; 3CD, protease precursor 3CD. B) Nucleotide similarity (percentage sequence identity) comparison for the 5′-untranslated region (UTR), capsid protein VP1, and protease precursor 3CD regions. Numbers along the x- and y-axes represent single serotypes. Operational taxonomic units are ordered according to their respective trees.
